# Appropriateness over Numbers: Pharmacological Burden and Scalable Medication Review in Older Adults

**DOI:** 10.3390/geriatrics11040100

**Published:** 2026-08-10

**Authors:** Michał Pstrągowski, Dawid Hachlica, Łukasz Izbicki

**Affiliations:** 1 Institute of Clinical Sciences, Maria Sklodowska-Curie Medical Academy in Warsaw, Plac Żelaznej Bramy 10, 00-136 Warsaw, Poland; michal.pstragowski@uczelniamedyczna.com.pl; 2 Medical Department, leki.pl Sp. z o.o., Rtm. Witolda Pileckiego 67/109, 02-781 Warsaw, Poland; dawid.hachlica@leki.pl; 3 Department of Drug Technology and Pharmaceutical Biotechnology, Faculty of Pharmacy, Medical University of Warsaw, Banacha 1, 02-097 Warsaw, Poland

**Keywords:** polypharmacy, older adults, deprescribing, potentially inappropriate medications, medication review, drug burden, prescribing cascade, clinical decision support

## Abstract

Polypharmacy in older adults is common, rising and inconsistently defined, and it is widely treated as a problem of how many medicines a person takes. This non-systematic narrative review argues that its harm depends more on the appropriateness of the regimen and the pharmacological burden the regimen imposes, with the number of drugs a partly confounded marker rather than the principal cause. Drawing on evidence published mainly since 2020, it traces the shift from counting medicines to weighting them; the prescribing cascade as a patterned and detectable source of inappropriate use; the explicit criteria (Beers, STOPP/START, EURO-FORTA) and implicit measures (the Medication Appropriateness and Drug Burden indices) that give appropriateness operational form; and the evidence that deprescribing is feasible and generally safe, although its benefit is conditional and depends on how it is done. Because these criteria can be applied to medication lists held in routine data, potentially inappropriate prescribing can increasingly be identified beyond the single encounter and at population scale, even as automated tools remain unreliable at judging whether a flagged criterion applies to the individual patient. The review concludes that medication review should prioritise appropriateness and the individual benefit–risk balance rather than the medication count, and that detection can increasingly be scaled even though the clinical decision and the patient’s agreement cannot.

## 1. Defining Polypharmacy: A Moving Target, and the Scale of the Problem

The ageing of populations worldwide has been accompanied by a rising burden of multimorbidity, and with it, the routine use of multiple medications [1,2]. Polypharmacy, understood broadly as the use of more drugs than a patient’s clinical circumstances warrant, has become one of the defining problems of geriatric pharmacotherapy, yet the term resists precise definition and it remains unsettled whether it is harmful in itself or chiefly a marker of underlying illness.

Definitional disagreement is extensive. A systematic review by Masnoon et al. identified 138 definitions of polypharmacy and associated terms across 110 studies, and a more recent count reached 143 [1,3]. The large majority are purely numerical, resting on a simple count of concurrent medications, with thresholds ranging from two or more to eleven or more drugs [1,3]. Across this heterogeneity, one cut-point recurs: the concurrent use of five or more medications is the single most common definition, applied in 46.4% of the studies reviewed by Masnoon, and it underpins the World Health Organisation’s working description of polypharmacy as the routine use of five or more medicines [3,4]. Ten or more drugs is widely labelled hyper- or excessive polypharmacy. The five-drug threshold is a pragmatic convention rather than a biological boundary. When Gnjidic et al. derived cut-points empirically, the number at which risk rose most sharply ranged from 4.5 drugs for mortality and falls to 6.5 for frailty, and no threshold predicted cognitive impairment [5].

A count of medicines indicates nothing about whether those medicines are warranted. Central to the modern understanding of the problem is the distinction between appropriate (or necessary) polypharmacy and problematic or inappropriate polypharmacy [1,6]. In the former, multiple drugs are justified by multimorbidity and prescribed in line with the evidence. In the latter, the cumulative harm outweighs the intended benefit. The distinction is rarely operationalised, and Masnoon et al. found explicit recognition of appropriateness in 6.4% of the included articles [3]. Later refinements add categories such as polypharmacy of unclear benefit and unnecessary polypharmacy [1,7]. The number of drugs is therefore a weak guide to the quality of prescribing.

This bears on whether polypharmacy causes adverse outcomes or instead identifies the patients most likely to experience them. The evidence so far supports association more than established causation, because prospective interventional data on the clinical impact of reducing drug counts remain limited [1,3]. Confounding by indication is the central difficulty, since patients on more medications also carry more disease. The findings diverge accordingly. In one large cohort, polypharmacy was no longer independently associated with non-cancer mortality once chronic conditions were accounted for and patients were matched by propensity score, which suggests the count was mainly marking underlying illness. In a nationwide Korean study and a Danish cohort, however, the association persisted after adjustment, with mortality rising by roughly 3% per additional drug [1].

Whatever its definition, polypharmacy is common, and reported prevalence varies widely with age, setting, region and the threshold applied [1]. The first meta-analysis of prevalence, by Delara et al., pooled 54 studies and estimated an overall prevalence of 37% (95% CI 31–43), with high heterogeneity between studies [8]. The figure rose with age and with the intensity of the care setting, reaching 45% (95% CI 37–54) in adults aged 65 or older and 52% (95% CI 38–66) in inpatients, against 20% in community samples [8].

**Figure 1:**
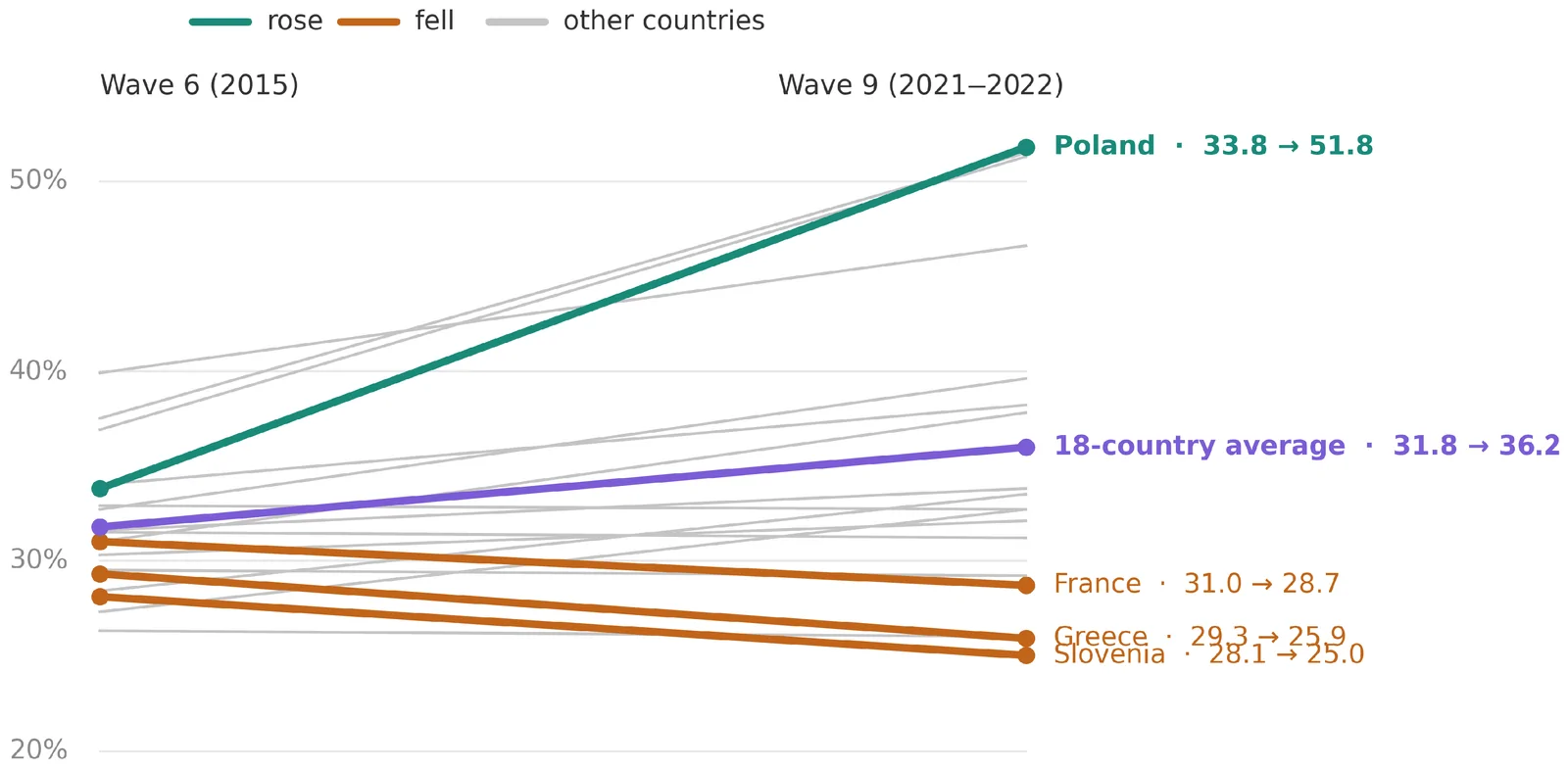
Prevalence of polypharmacy (five or more medicines) among adults aged 65 or older in the SHARE survey, by country, between wave 6 (2015) and wave 9 (2021–2022). Each line is one country; the 18-country average, Poland (rising), and France, Slovenia, and Greece (falling) are highlighted, with the remaining countries shown in grey. Because the estimates derive from a single source with one definition and are standardised for age and sex, levels are comparable across countries; the direction of change differed, with 11 of the 18 countries and regions covered by both waves (17 European countries and Israel) rising and 7 falling. Values for both waves are taken as published in [11], where they are standardised for age and sex to the 2013 European standard population. No data were read from published figures and no reanalysis was performed, other than the unweighted average across the countries common to both waves. The figure is an original visualisation prepared by the authors and is not a reproduction or adaptation of any figure in the cited source, which is published under a CC BY licence.

National time-series data show a rising trend across very different health systems. In the United States, successive National Health and Nutrition Examination Survey cycles recorded a rise in polypharmacy (five or more prescription drugs) among all adults, from 8.2% (95% CI 7.2–9.2) in 1999–2000 to 17.1% (95% CI 15.7–18.5) in 2017–2018 [9]. Among adults aged 65 or older, the figure reached 44.1% [9]. In Europe, the Survey of Health, Ageing and Retirement in Europe (SHARE) found an overall prevalence of five or more drug use of 32.1% (95% CI 31.5–32.7) in wave 6 [10]; by wave 9 (2021–2022), the age- and sex-standardised figure had risen to 36.2%, ranging from 25.0% in Slovenia to 51.8% in Poland [11]. The direction of change between the two waves varied across countries (Figure 1). National series that cover longer periods show steeper increases. In Ireland between 1997 and 2012, polypharmacy rose from 17.8% to 60.4% and excessive polypharmacy from 1.5% to 21.9% [12]. In Tayside, Scotland, between 1995 and 2010 the proportion dispensed ten or more drugs tripled to 5.8% and the proportion dispensed five or more doubled to 20.8% [13].

Prevalence is consistently highest in institutional and acute settings: 44% in a Swedish register-based cohort [14], close to 50% on five to nine drugs (with a further 24.3% on ten or more) among European nursing-home residents [15], and as high as 96.5% (hyper-polypharmacy, eleven or more drugs) among hospitalised patients aged 80 or over in China [16].

This range should be read with caution, because much of the variation arises from how polypharmacy is defined and recorded rather than from real differences between populations. In the systematic review by Nicholson et al., a five-drug cut-point was used in 77% of studies, yet only 25% specified which medicines were actually counted, for example whether over-the-counter drugs and supplements were included [2]. The same drug count can therefore mean different things from one study to the next. Most studies rely on a bare numerical count applied to heterogeneous data sources, which limits comparability across datasets. Without transparent, harmonised definitions, polypharmacy is hard to measure consistently across populations, and consistent measurement underpins both surveillance and any systematic attempt to reduce it. The same caution applies to the direction of change in Figure 1, since the two waves are separate cross-sections rather than a cohort followed over time, and the estimates are self-reported [11]. Why some countries fell while most rose is taken up in Section 9.

Pharmacological burden is the cumulative exposure a regimen imposes, weighted by the pharmacological activity of its components rather than by their number. It is continuous, dose-dependent and calculable from dispensing records, and the Drug Burden Index is its most widely used expression [17]. Potentially inappropriate prescribing describes a prescription that meets an explicit criterion identifying it as more likely to harm than to benefit older adults as a group. Such a criterion flags a prescription for review without settling it, and the qualifier “potentially” records that limit. Medication appropriateness is the judgement of whether a particular medicine is right for a particular patient, given the indication, the balance of benefit and harm for that person, and their care goals, prognosis and preferences [18]. Burden is a property of the regimen and can be measured, potential inappropriateness is a property of a prescription assessed against a rule and can be computed, and appropriateness is a property of a clinical decision about an individual patient and can be neither.

**Figure 2:**
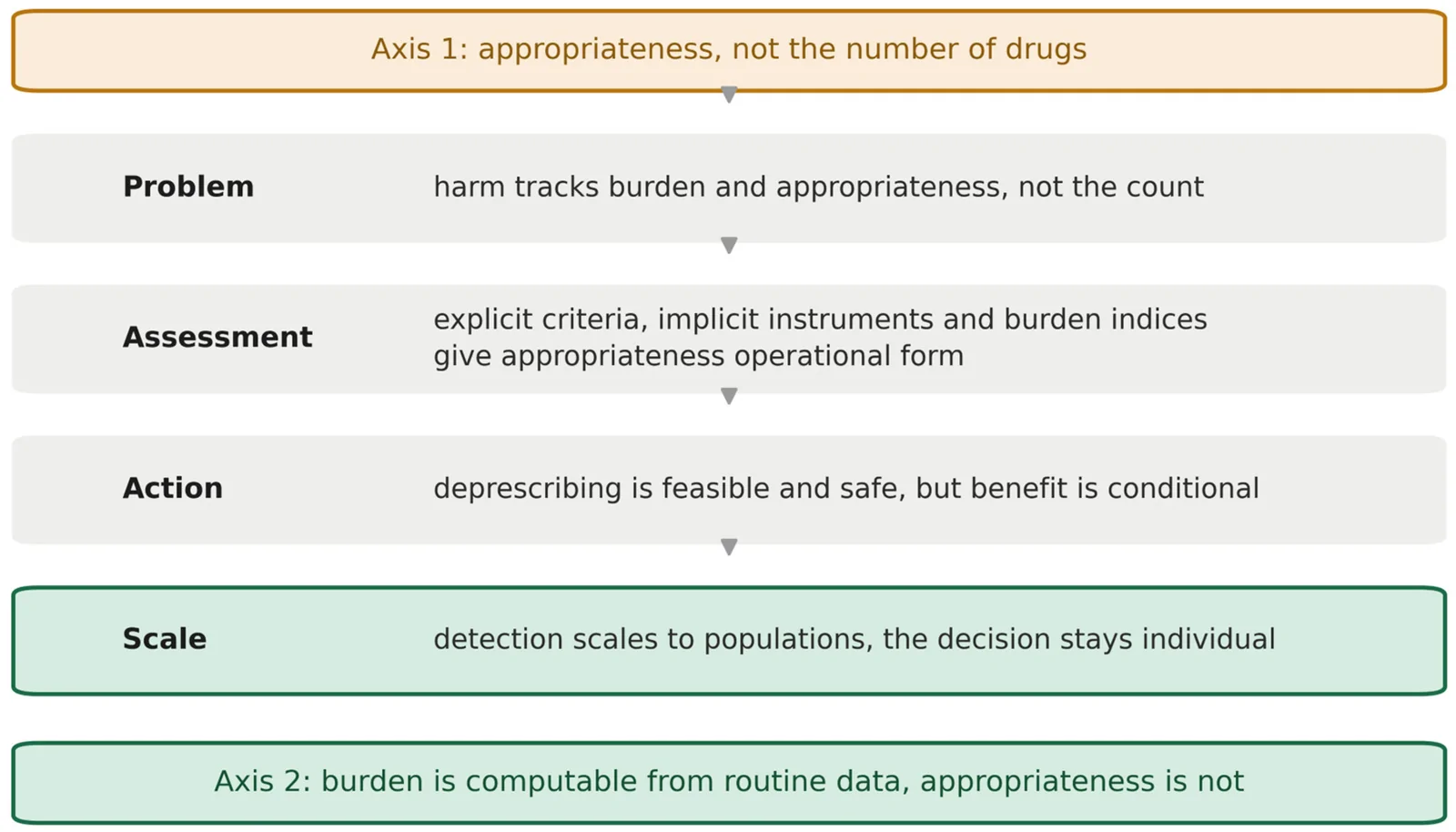
The four-phase logic of this review: the problem (what the harm is), assessment (how to detect it), action (what to do) and scale (how to deliver it). Two axes run throughout: the appropriateness and pharmacological burden of a regimen (Axis 1), and the extent to which burden and potentially inappropriate prescribing can be computed from routinely collected data, which allows detection to scale from the bedside to whole populations (Axis 2).

Since 2020, the field has accumulated new evidence: the principal prescribing criteria have been revised, the largest deprescribing syntheses have been published, and medication review has been attempted computationally and across whole populations. This review advances two linked claims. The first is that the harm of polypharmacy turns on the appropriateness and pharmacological burden of a regimen more than on the number of medicines, although the number contributes in its own right. The second is that pharmacological burden, and some criteria for potentially inappropriate prescribing, can increasingly be computed from routinely collected data, while appropriateness itself cannot. On that basis, it asks how potentially inappropriate prescribing might be identified and acted on beyond the single clinical encounter and across whole populations (Figure 2).

## 2. Methods: Literature Search and Selection

This article is a narrative review, not a systematic or scoping review, and does not follow a protocol-driven methodology. The literature was identified through searches of PubMed, Embase and Google Scholar carried out between January and June 2026, supplemented by hand-searching the reference lists of relevant articles. Search terms combined the core concept of polypharmacy with terms for older adults and for the specific topics addressed in each section, among them drug burden, prescribing cascade, potentially inappropriate prescribing, deprescribing, medication review and clinical decision support. Terms were combined and refined as relevant literature emerged, without a predefined set of queries. Priority was given to evidence published between 2020 and 2026, with foundational or definitional works cited irrespective of date where they remain standard points of reference.

Only English-language sources were considered. Conference abstracts, preprints, case reports, small case series and sources whose full text could not be obtained were not cited. Reports from international and national bodies were included where they are the standard reference on a point. Where more than one source addressed the same point, systematic reviews and meta-analyses were preferred, followed by large cohort studies with full adjustment for confounding. For questions of intervention, the evidence was drawn from randomised trials where these were available. Multinational and national data were used ahead of single-centre studies. The review was self-assessed against the Scale for the Assessment of Narrative Review Articles (SANRA) [19], and the completed form is provided as the Supplementary Materials.

Selection was purposive rather than exhaustive: sources were chosen to represent the main positions and the strongest evidence on each question, including findings that complicate the review’s argument as well as those that support it. Where the evidence on a question was divided, both positions were reported, and the conclusions of authors who read the same data differently are given in their own terms. No formal risk-of-bias assessment was undertaken. Where the authors of a pooled analysis reported heterogeneity or the certainty of their findings, these are given alongside the estimate. As with any narrative review, this approach does not guarantee comprehensive coverage and is subject to selection bias. The search was neither protocol-driven nor registered, so it cannot be reproduced step by step. The restriction to English will have excluded relevant work. The weight given to individual studies rests on the authors’ reading rather than on a scored appraisal. A further limitation lies in the evidence rather than in the method. Most of it comes from high-income health systems, the prevalence estimates are largely cross-sectional and self-reported, and few deprescribing trials recorded frailty at baseline, so how far these findings transfer to other health systems and to frailer patients is unknown. The synthesis is therefore an interpretive overview and not a systematic appraisal of all available evidence.

## 3. From Counting to Weighting: The Pharmacological Burden

A count records how many drugs a patient takes, not what those drugs are. Age-related changes in drug handling are well described, but the more basic limitation is that drugs are not interchangeable. Two patients each taking five medicines may carry very different pharmacological loads, because a simple count weights a vitamin supplement and a sedative–hypnotic equally [1].

The Drug Burden Index (DBI), proposed by Hilmer et al., captures this difference by weighting cumulative exposure to anticholinergic and sedative medicines according to dose, on the principle that these two pharmacological actions account for much of the functional harm attributed to medication in older adults [17]. In the Health, Ageing and Body Composition cohort (n = 3075, aged 70–79 years), each one-unit increase in DBI was associated with a 0.15-point lower score in physical function (*p* < 0.001) and a 1.5-point lower score on the Digit Symbol Substitution Test (*p* = 0.01). These effects were comparable in magnitude to three and four additional comorbidities, respectively [17]. The total number of medications lacking anticholinergic or sedative activity was not associated with poorer function [17]. The DBI can also be calculated directly from prescribing data through a defined dose–response formula.

A second quantifiable dimension is the potential for drug–drug interactions, which rise disproportionately as more drugs are added. Among 404 older inpatients with cardiovascular disease, taking an average of 11.6 medications, 77.5% had at least one severe potential interaction on screening [20]. Such interactions are detectable by rule against a drug list, but the same study shows the limits of rule-based detection. The concurrent use of an antiplatelet and an anticoagulant is flagged as serious, yet is appropriate in selected patients with ischaemic heart disease and atrial fibrillation, leading the authors to caution that interaction-screening programmes cannot replace clinical judgement [20].

A count of prescriptions also leaves out what patients take on their own. In a cohort of more than 15,000 community-dwelling older adults in Australia and the United States, 66.2% reported using at least one dietary supplement or complementary medicine in the previous month, most often vitamin D, fish oil and calcium [21]. In a United States cohort whose regimens were recorded by brown-bag review rather than by questionnaire, 84% used at least one supplement over two years, and supplements accounted for around 30% of the total pill burden [22]. Individual herbal products were used far less often than vitamins and minerals in that cohort, with turmeric the most common [22]. Both cohorts were healthy and comparatively advantaged, so these proportions are unlikely to hold across all older populations. These products are bought without a prescription and therefore leave no trace in dispensing records, although surveys that ask patients directly do capture them, and the SHARE question behind the European prevalence figures counts drugs bought without a prescription and dietary supplements alongside prescribed medicines [11]. Two patients on the same number of drugs can therefore carry very different burdens, and part of the difference is not in the record at all. Asking the patient to bring in everything they take, including what they buy themselves, adds information that no dispensing dataset contains. A particularly important route by which this burden accumulates is the prescribing cascade.

## 4. The Prescribing Cascade: A Sequential, Detectable Pattern

The medication burden can also grow sequentially, as one drug leads to another. In a prescribing cascade, the adverse effect of one drug is taken for a new illness and treated with a second, so that the harm of the first is compounded by the risk of the second [23]. First described three decades ago, the concept is now recognised as an important and under-appreciated dimension of problematic polypharmacy and a useful starting point for medication review [6,23]. A cascade usually operates at the level of drug classes, not individual agents. The long-established link between non-steroidal anti-inflammatory drugs and the initiation or intensification of antihypertensive therapy can involve any of some twenty non-steroidal drugs and more than forty antihypertensives on a single formulary [23]. A second, widely cited example is the use of a cholinesterase inhibitor for dementia followed by an anticholinergic to treat the resulting urinary incontinence, in which two drugs work at cross purposes [23]. The concept is not confined to prescriptions. Rochon and Gurwitz extend it to adverse effects managed with over-the-counter products, supplements or devices, giving the example of a cholinesterase inhibitor whose gastrointestinal effects are self-treated with bismuth subsalicylate, and of one whose bradycardia ends in a pacemaker [23]. Cascades of that kind leave no trace at all in dispensing records.

Prescribing cascades can be detected at the population level because they unfold sequentially, and that sequence is recorded in prescribing data. Prescription sequence symmetry analysis (PSSA), a pharmacovigilance method that compares how often drug B is started after drug A versus before it, can detect cascades at the level of populations. Applied to 1.2 million new users of a dihydropyridine calcium-channel blocker, it identified an excess of loop-diuretic initiation consistent with treatment of drug-induced oedema (adjusted sequence ratio 1.87, 95% CI 1.84–1.90), with the effect concentrated in the first four months and rising with the starting dose [24]. In absolute terms, the authors estimated that 1.4% of those starting the drug experienced the cascade within a year, and about one in nine of those who developed oedema. The incidence was twice as high at 65 years and over as below it, 2.3% against 1.1% [24].

The same cascade has since been replicated in independent national datasets in the Netherlands (adjusted sequence ratio 1.82, 95% CI 1.77–1.86) and Ireland (1.93, 95% CI 1.79–2.09), the latter restricted to adults aged 65 or older [25,26]. A systematic review of this approach identified 101 studies, 77% of which reported at least one positive signal [27]. In the same Dutch analysis, an expert panel judged 66 cascades potentially problematic, and PSSA then found a statistically significant signal for 41 of them, the strongest being amiodarone followed by thyroid hormone for drug-induced hypothyroidism (adjusted sequence ratio 4.63, 95% CI 4.40–4.85) [25]. Several of these cascades are dose-dependent, and polypharmacy itself increases their likelihood, since the more drugs a patient takes, the harder an adverse reaction is to recognise as such [27,28].

That a cascade is detectable in data does not mean it is reliably identifiable in an individual. PSSA cannot establish causation, is prone to false-positive and false-negative signals, and says nothing about intent: from dispensing records alone, an appropriate cascade cannot be distinguished from an inappropriate one [27], nor is a cascade always a mistake. Chart review has found prescribers who had recorded the oedema as drug-induced and added a diuretic anyway, sometimes immediately after reducing the offending dose, and the literature now distinguishes intentional cascades from unintentional ones [26,27,29]. When the flagged cases from one such signal were checked against medical records, the loop diuretic had been started for calcium-channel-blocker oedema in 35 of 64 patients, a positive predictive value of 55%. Removing those confirmed cases restored symmetry to the sequence (adjusted sequence ratio 1.05, 95% CI 0.62–1.78), so the population-level signal held even though the flag identified the right patient only about half the time [29]. A sequence in prescribing data should therefore be treated as a trigger for clinical review, not as proof of an inappropriate cascade. For the clinician, the practical question is what to do once a sequence is suspected, and Rochon and Gurwitz set out three [23]. Before treating a new symptom, ask whether it could be an adverse effect of a drug the patient is already taking. If a cascade is identified, ask whether the initial drug is still needed, and whether a safer alternative or a lower dose would remove the reason for the second. Then, weigh the harms and benefits of continuing the initial drug with the patient rather than for them. The second drug is usually the one that prompts the review, although it is the first that has to be reconsidered if the cascade is to be undone.

## 5. Clinical Consequences of Polypharmacy

Pharmacological burden and prescribing cascades are clinically important because they are associated with adverse outcomes in older adults. Polypharmacy has been linked to a broad spectrum of them: higher mortality, hospitalisation, adverse drug reactions, impaired physical function, and injurious falls. These associations are well established, but their causal interpretation is contested. Because polypharmacy is most often the downstream consequence of multimorbidity, the number of medications is closely entangled with the disease burden that prompts prescribing in the first place. Confounding by indication, therefore, affects every estimate, and the better-adjusted studies repeatedly find that associations attenuate, sometimes considerably, once comorbidity and the use of specific high-risk drug classes are taken into account.

A meta-analysis of 24 cohorts (almost three million participants) reported a graded association between polypharmacy and all-cause mortality, with pooled risk rising from 1.28 (95% CI 1.19–1.39) at a threshold of five or more medications to 1.44 (95% CI 1.03–2.01) at ten or more, alongside a comparable increase in hospitalisation (1.37; 95% CI 1.09–1.71) [30]. The authors were explicit, however, that heterogeneity was very high (I^2^ = 94.7%), that confidence in the evidence was low by GRADE, and that causality remained unclear [30].

The Moli-sani study, following community-dwelling adults aged 65 or older with time-varying exposure and inverse-probability weighting, similarly found higher mortality (hazard ratios 1.21–1.30) and hospitalisation (1.39–1.61) [31]. Part of this excess was statistically mediated by potentially inappropriate prescribing, although the mediated share was modest, an estimated 13.6% of the effect on mortality and 6.0% on hospitalisation. Notably, the risk was similar in older adults without multimorbidity, the group in whom multiple medications are least obviously warranted [31]. The harm persisted in those without multimorbidity and was only partly mediated by potentially inappropriate prescribing, a pattern that implicates the pharmacological burden and appropriateness of the regimen more than the number of medications.

The clearest direct link between multiple medications and harm is the adverse drug reaction. In a prospective study of acute medical admissions, adverse drug reactions were present in 18.4% of patients and were judged the cause of 16.5% of admissions. Affected patients were taking more medications, a mean of 10.5 versus 7.8 [32]. Two findings are particularly relevant to medication review. Around 40% of these reactions were considered avoidable, and the large majority (86.2%) were type A reactions: dose-dependent, predictable extensions of known pharmacology, and therefore foreseeable through review [32]. Nearly a third (29.4%) involved a drug–drug interaction [32], tying this outcome directly to the countable interaction burden.

The mechanisms behind these figures are visible in everyday combinations. Anticholinergic and sedative drugs accumulate without any single prescription looking unreasonable, since each is started for its own indication, and it is the summed exposure that carries the functional and cognitive cost [17]. Renal injury arises differently, from a combination rather than from accumulation. Among 487,372 users of antihypertensive drugs, adding a non-steroidal anti-inflammatory drug to a diuretic together with an angiotensin-converting enzyme inhibitor or an angiotensin receptor blocker was associated with a higher rate of acute kidney injury (rate ratio 1.31, 95% CI 1.12–1.53), concentrated in the first 30 days of the combination (1.82, 1.35–2.46). In that study, neither two-drug combination carried the same excess [33]. Bleeding shows a third pattern, in which the interaction is recognised and the decision is still difficult, as when an antiplatelet is combined with an anticoagulant in a patient who has both ischaemic heart disease and atrial fibrillation [20]. Each of these is the kind of predictable, dose-dependent reaction that accounts for the large majority of drug-related admissions [32].

Older adults carry these risks disproportionately, and part of the reason precedes the prescription. Ageing alters both the handling of a drug and the response to it, so the same dose need not produce the same exposure or the same effect at eighty as at fifty [34]. Liver volume and hepatic blood flow fall, which raises the bioavailability of drugs subject to extensive first-pass metabolism, and the decline in glomerular filtration rate slows the clearance of drugs eliminated by the kidney. Reduced muscle mass conceals part of that decline, since serum creatinine can sit within the reference range while renal function is markedly diminished [34]. Sensitivity to drugs acting on the central nervous system rises at the same time, so that sedation, postural sway and memory impairment are more pronounced at a given benzodiazepine dose, and in dementia, the loss of cholinergic transmission compounds the central effects of anticholinergic drugs [34]. Impaired homeostatic mechanisms, among them reflex tachycardia and the regulation of temperature and electrolytes, add further to the risk of an adverse drug reaction. Changes in vision, hearing, swallowing and cognition bear on whether a regimen is taken as intended [34].

The number of medicines also bears on whether a regimen is taken as prescribed. A meta-analysis of twelve studies using the Medication Regimen Complexity Index found greater complexity associated with non-adherence (adjusted OR 1.05, 95% CI 1.02–1.07) and with first hospitalisation (HR 1.20, 95% CI 1.14–1.27), though not with readmission after adjustment (OR 1.04, 95% CI 0.93–1.16) [35]. The authors describe the effect on adherence as small and report that they could not adjust for the number of medicines, which is associated with the same outcomes [35].

Beyond discrete events, polypharmacy has been associated with gradual functional decline. In the HILDA cohort, incident polypharmacy was accompanied by the steepest deterioration in physical and mental summary scores on the 36-Item Short Form Health Survey (SF-36), and the authors stressed that reducing the count does not in itself guarantee benefit. What is required is a review of whether each medication still serves the patient [36]. The cognitive evidence points the other way. In a large cohort of older adults with incident dementia, the number of medications did not predict the rate of decline in Mini-Mental State Examination scores after extensive adjustment for confounders [37]. The authors concluded that measures of pharmacological burden, such as the anticholinergic and sedative load captured by the Drug Burden Index, are more informative for predicting cognitive decline than the number of drugs alone.

Evidence on falls illustrates this tension particularly clearly, and is now substantial and consistent across countries and methods. A Swedish nationwide nested case–control study of 49,609 injurious-fall cases found that risk rose with each additional drug in a near-linear, consistent fashion (odds ratio 1.02 per drug; 95% CI 1.01–1.03), which in principle supports a causal reading. Yet after full adjustment for fall-risk-increasing drugs (FRIDs) and multimorbidity, the association was, in the authors’ words, substantially weaker than previously reported, and the corresponding population-attributable fraction was only 5.2% (95% CI 2.8–7.6). Even if the relationship were wholly causal, eliminating the excess attributable to polypharmacy would prevent roughly one injurious fall in twenty [38].

The absolute scale of this risk is informative in other cohorts too. In a Quebec cohort of over 1.1 million older adults, fall-related hospitalisation occurred in 1.8% of women and 1.1% of men over a single year. The adjusted hazard ratio reached 2.17 (95% CI 2.05–2.31) for ten or more medications. Yet the authors framed this as practical risk stratification rather than causal attribution, and showed that a simple count performed no worse than dedicated FRID or potentially inappropriate medication tools, while clinical data outperformed all three [39].

In the English Longitudinal Study of Ageing, analysed with competing-risk regression and adjusted for both multimorbidity and FRIDs, the proportion hospitalised for a fall rose stepwise with medication use, from 1.5% among those taking none to 4.7%, 7.9% and 14.8% across successive categories. The risk was concentrated at the extreme (subdistribution hazard ratio 3.19; 95% CI 1.61–6.32, for ten or more medications) [40].

The interpretation of these findings remains contested. The ELSA investigators concluded that prescriptions should be reviewed regularly and the number kept to a minimum, a defensible position given that every avoidable medication is a modifiable exposure and that the excess risk at ten or more drugs persisted despite adjustment for both FRIDs and multimorbidity. The Swedish and Canadian analyses reach a more cautious conclusion: that the count is a confounded marker, useful for stratification but weak as a target in itself. The natural-experiment evidence tends to favour caution. A policy that reduced benzodiazepine use in New York State by roughly 60% produced no corresponding fall in hip fractures [41]. The prescribing cascade suggests the same: it is particular agents, not the aggregate number, that cause harm. Prochlorperazine prescribed for dizziness, for example, has been associated with a subsequent increase in hip fracture [27].

The consequences of polypharmacy are consistent across studies, yet their magnitude shrinks under proper adjustment and their causal status is only partial. Some of what remains runs through the number of medicines itself, by way of interaction risk, regimen complexity and adherence. This evidence makes the case for identifying and addressing inappropriate polypharmacy through structured review, not for reflexively reducing the number of medications.

## 6. Tools for Identifying Potentially Inappropriate Prescribing

Identifying inappropriate polypharmacy raises an operational question. How is appropriateness to be judged? Over four decades, the response has been a succession of explicit tools, criteria and lists that name the medications best avoided in older adults, complemented by implicit, judgement-based instruments such as the Medication Appropriateness Index [42,43] and by the pharmacological burden measures, of which the Drug Burden Index is the clearest example [17]. Explicit criteria have come to dominate everyday practice and research because they are reproducible and, unlike a bedside judgement, can be applied directly to a list of dispensed medicines (Table 1).

Two systems anchor the field. The Beers Criteria, maintained by the American Geriatrics Society since 2011 and updated most recently in 2023, are an explicit list of medications typically best avoided by adults aged 65 or older across ambulatory, acute and institutional settings, organised into categories that include drugs to avoid outright, drug–disease and drug–drug interactions, and agents requiring caution or dose adjustment [44]. They are, by design, specific to United States practice. The European counterpart, the STOPP/START criteria, reached its third version in 2023 and is broader in one respect: alongside 133 STOPP rules flagging potentially inappropriate medications, it contains 57 START rules flagging the opposite error, namely potential prescribing omissions, beneficial drugs that should have been started but were not [45]. The expansion to 190 criteria, two-thirds more than the previous version, reflects both accumulating evidence and the breadth of the problem [45]. The START criteria matter because inappropriate prescribing includes underuse as well as overuse.

Beyond these two, a proliferation of region-specific instruments now exists, reflecting differences in national formularies and prescribing cultures. The EURO-FORTA list, validated in a second version across seven European countries or regions, classifies 267 drug entries across 27 indications by clinical appropriateness from A to D, labelling beneficial as well as inappropriate agents rather than cataloguing only what to avoid [46]. PRISCUS 2.0, the updated German list, classes 187 substances as potentially inappropriate [47], against a background in which the proportion of German adults aged 65 or older receiving at least one such drug fell from roughly a quarter in 2009 to 14.5% by 2019 [47]. STOPPFrail version 2 narrows the focus to deprescribing in frail older adults approaching the end of life, offering 25 criteria built explicitly around shared decision-making [48]. The frequency of potentially inappropriate prescribing bears out the need for this much instrumentation: a meta-analysis of 94 studies, covering nearly 371 million older adults across 17 countries, estimated a pooled prevalence of potentially inappropriate medication use of 36.7% (95% CI 33.4–40.0), ranging from 23.6% in Oceania to 47.0% in Africa [49]. Because these tools are rule-based, they are well suited to electronic implementation, although how much of a criterion set can be applied depends on what the data hold. Criteria that turn on drug–drug interactions, therapeutic duplication or treatment duration can be derived from dispensing records alone. Those that turn on a diagnosis, a laboratory result, a treatment indication, an expected prognosis or a patient’s preference require clinical data that dispensing records do not hold. No single list is definitive, and the realistic standard is regional validity rather than universality.

For all their utility, these instruments flag prescriptions for review rather than confirm errors, and their real-world yield is low. The criteria’s own developers report that the proportion of software-generated STOPP/START recommendations actually implemented has been modest, low in pragmatic trials and higher only with intensive, face-to-face pharmacist review. In one large trial, more than half of the recommendations generated by software were judged clinically unimportant once the individual patient was considered [45]. The authors are explicit that the criteria are an aid to clinical judgement and cannot replace it [45]. This echoes the prescribing-cascade literature, in which only around half of the signals flagged in dispensing data proved genuine on review. As Petrovic, O’Mahony and Cherubini put it, inappropriate prescribing is a matter of both hazards and solutions [50].

The choice between these instruments depends on the question asked and on the data at hand. For surveillance and population screening, the explicit criteria are the practical option, because the parts of them that rest on the medication list alone can be coded without clinical contact. Beers and STOPP/START serve that purpose in the United States and Europe, while national lists such as PRISCUS 2.0 match their own formularies more closely. The Drug Burden Index suits the same setting, and it has been applied to larger populations than any other measure of anticholinergic burden, mostly through database studies [51]. Its use in day-to-day care is constrained by the time it takes to calculate and by restricted access to the Drug Burden Index Calculator [51]. For the review of an individual patient, the implicit instruments carry more information, and the Medication Appropriateness Index rates indication, effectiveness, dose and duration drug by drug, at the cost of rater time and rater variability. EURO-FORTA needs the indication to be known, which suits a clinical encounter better than a dispensing record, and STOPPFrail version 2 is the closer fit in advanced frailty.

Whether any of these instruments predicts clinical outcomes better than the others has not been established. Among the anticholinergic burden scales, only two studies have compared eight scales simultaneously; the findings for delirium, cognition, mortality and falls conflict, and none of them is treated as a standard [52]. Comparisons between the explicit criteria and the burden measures are scarcer still. Instrument choice therefore follows the setting and the data available.

For a single patient, the instruments are used together, and the order follows from the data available. The dispensing record alone supports the criteria that turn on interactions, therapeutic duplication and treatment duration, together with the Drug Burden Index. Anything resting on a diagnosis, a laboratory value, an indication or a prognosis needs the clinical record. Apply the criteria set that matches the national formulary, since a drug flagged in one country may not be marketed in another [44]. Screen for omissions in the same pass, because the START criteria address undertreatment alongside the drugs best avoided. Where anticholinergic and sedative drugs have accumulated, a burden measure records what a count of flagged criteria does not [17]. In advanced frailty or limited life expectancy, STOPPFrail version 2 is the shorter and better-matched list [48]. A flag marks a candidate for review, and whether it applies to this patient is a clinical judgement that no criterion set can make for the clinician [45].

Detection alone is not enough. Turning a flagged medication into a safe change is the work of structured medication review and deprescribing. Applying these criteria across whole populations, rather than one patient at a time, is where their computability finally pays off.

## 7. Deprescribing and Structured Medication Review

These instruments identify potentially inappropriate medicines; the harder question is whether changing them improves patient outcomes. Deprescribing is the structured process of tapering or stopping drugs whose harms or burdens outweigh their benefits within an individual’s care goals, functional status and life expectancy. It is a deliberate, patient-centred intervention requiring shared decision-making and close monitoring, not the simple withdrawal of treatment [18]. Scott and colleagues set out a five-step protocol: ascertaining every drug and its indication, gauging each patient’s overall risk of drug-induced harm, weighing each drug’s benefit against its harm or burden, prioritising those with the least favourable balance, and implementing withdrawal one drug at a time with monitoring [18]. The case is clearest near the end of life and in advanced frailty, where preventive drugs are least likely to confer benefit within remaining lifespan and where dedicated instruments such as STOPPFrail support shared decisions [48]. The rationale has often rested on the premise that fewer drugs mean less harm, and the trial evidence tests that premise directly.

That evidence is now substantial. The largest synthesis to date pooled 259 studies in more than 300,000 older adults and found that deprescribing to reduce polypharmacy did not significantly change mortality (OR 0.96, 95% CI 0.84–1.09), falls (0.88), fractures (0.97), adverse drug events (1.11), emergency presentations (0.85) or unplanned hospital admissions (0.99). Adverse drug-withdrawal events were not significantly increased, though the estimate was imprecise and drawn from three studies (OR 2.29, 95% CI 0.60–8.77), and the authors conclude that deprescribing is feasible and safe [53]. The pivotal randomised trial of structured review agrees on the hardest endpoint: in 2008 multimorbid inpatients, a doctor–pharmacist review supported by STOPP/START software reduced potentially inappropriate prescribing but left drug-related hospital admissions at twelve months essentially unchanged (21.9% versus 22.4%; HR 0.95, 95% CI 0.77–1.17), with no detriment to patients and only a modest gain in quality of life [54].

Where benefit is observed, it depends on how the intervention is targeted and delivered. In the same synthesis, subgroup analyses of the randomised studies found lower mortality when interventions were patient-specific (OR 0.79, 95% CI 0.63–0.99) and in those aged 65–79 (0.71, 0.51–0.99), whereas generalised approaches such as practitioner education or computerised decision support delivered without individual tailoring showed no effect (1.04, 0.81–1.33). Both estimates that reached significance did so with an upper confidence limit of 0.99. Benefit appeared greatest when review began early [53].

These subgroup findings should be read against the composition of the evidence pooled. The synthesis draws on 259 studies in more than 300,000 people, of which 95 were randomised, 50 were non-randomised with a concurrent control group and 114 had no concurrent control. Just over half were set in the community, the rest were divided between hospitals and residential aged care, and the weighted mean follow-up was 15.1 months. In 23 of the 37 randomised trials of deprescribing polypharmacy, the withdrawal schedule was not described. The authors report an I^2^ of 3% for the mortality estimate, while noting that heterogeneity remained in follow-up duration, withdrawal plan and intervention type, and that mortality was a secondary outcome in some trials not powered to detect a difference [53]. A separate appraisal of the deprescribing literature makes the point that a low I^2^ shows only that variation between studies is no greater than chance would predict, and says nothing about heterogeneity in the populations recruited, the medicines withdrawn or the interventions delivered [55].

Trials focused on single drug classes help explain this. Direct patient education led 27% of long-term benzodiazepine users to discontinue, against 5% under usual care (risk difference 23%; number needed to treat 4), an effect undiminished by concurrent use of ten or more drugs and free of serious harm. Even so, benzodiazepines were sometimes replaced by equally hazardous sedatives, and prescribers sometimes discouraged cessation [56]. Withdrawing one antihypertensive agent in patients over 80 was non-inferior for blood-pressure control and sustained in two-thirds, but carried a measurable physiological cost (systolic pressure higher by 3.4 mm Hg) and more adverse events (RR 1.28) [57]. Withdrawing fall-risk-increasing drugs did not reduce falls, and no trials reported fall-related fractures [58].

At the regimen level, the findings are consistent: deprescribing reliably lowers the number of medicines and the count of potentially inappropriate ones. Yet it does not reliably change the proportion of patients carrying at least one such drug (OR 0.87, 95% CI 0.75–1.02) or the Drug Burden Index (mean difference −0.08, −0.35–0.19) [53]. What benefit exists depends on individualised, early review and on avoiding the substitution of one harm for another. Such review is labour-intensive: the OPERAM intervention required roughly 97 min per patient from a doctor and pharmacist [54], whereas the low-touch, generalised tools that could scale showed no effect on outcomes [53]. Delivered one patient at a time, careful medication review cannot reach the populations in which potentially inappropriate prescribing is most prevalent. The unmet need is therefore a way to deliver targeted, patient-specific review to whole populations.

## 8. Automating Medication Review at Population Scale: Artificial Intelligence as a Complement to Clinical Judgement

Targeted, patient-specific medication review is hard to deliver at population scale because it depends on clinical judgement for each patient. Several of its components, however, can now be derived directly from routinely collected data. The explicit criteria have been operationalised for entire administrative populations: STOPP/START and Beers have been coded against linked health databases covering the older residents of a Canadian province, which allows potentially inappropriate prescribing to be enumerated across a whole population rather than chart by chart [59]. Automated detection performs well where the task is structured. A multilabel classifier developed on prescribing data from Chinese outpatients in tertiary hospitals flagged inappropriate medications against the Beers list with high accuracy on that dataset (best model: accuracy 97.8%, F1 score 0.91, on a 0–1 scale where 1 is perfect). The authors note, however, that criteria framed for one country transfer imperfectly to another, and that the absence of laboratory and clinical detail limits what such a tool can detect [60]. Combined with drug-burden indices calculable from dispensing records alone, the raw materials for a population-scale screen are in hand.

Deploying decision support at scale has not by itself improved hard outcomes. The pivotal trial of software-supported review left drug-related hospital admissions unchanged [54]. The largest synthesis found that generalised interventions, namely practitioner education and computerised decision support delivered without individual tailoring, had no effect on mortality, in contrast to patient-specific review [53].

Detecting an inappropriate medication is mechanical, but predicting who is at risk, and judging whether withdrawal suits a given person, requires clinical judgement. Tested across more than 1.1 million older adults, no machine-learning model, including gradient boosting, random forests and neural networks, outperformed ordinary logistic regression at predicting inappropriate medication use, with discrimination plateauing at an area under the curve of about 0.62. The single most informative predictor, where it was included, was simply the number of medications [61]. Routinely held structured data carry only a modest predictive signal.

A single retrospective study of a large language model shows the same limitation. Applied to older emergency-department patients with polypharmacy, a GPT-4o pipeline filtered the relevant deprescribing criteria for each medication list more accurately than trained junior reviewers (F1 0.86) [62]. At the next step, deciding whether a flagged criterion actually applied to the individual, performance fell sharply (F1 0.58), with errors driven by complex inclusion and exclusion conditions and by missing clinical information. The model over-recommended withdrawal, in one instance suggesting that anticoagulation be stopped in a patient with recent thromboembolism, and its stated confidence aligned poorly with its accuracy [62]. These figures describe one model applied once to one clinical population, and how they would transfer to other settings has not been tested.

These findings support a triage model and define its limits. Such a screen would stratify a population, with the decisions left to clinicians. Using computable burden measures, it identifies the patients most likely to benefit from review and refers them for targeted, patient-specific assessment. The evidence that such assessment lowers mortality comes from a subgroup analysis rather than from the primary comparison, which found no effect [53]. That assessment cannot be delivered to a whole population by hand, since it takes roughly 97 min of clinician and pharmacist time per patient [54]. Reviews of artificial intelligence in this setting reach the same qualified conclusion. Such tools accelerate detection and may lower cost, but evidence that they improve morbidity, mortality or quality of life is so far absent, and they are best understood as a complement to clinical judgement under human oversight, not a substitute for it [63]. The evidence in this area rests on a small number of recent studies, each in a single healthcare system, and should be read as preliminary. Table 2 sets out what such a sequence would require at each stage, who would carry it out and what it cannot do. The first two stages could be implemented with any measure computable from dispensing records, and the framework does not depend on a particular instrument or system. It is offered as a proposal to be tested rather than as a specification for implementation.

## 9. Barriers and Facilitators: The Patient’s Role

Potentially inappropriate prescribing can be defined, detected increasingly from routine data, and, where appropriate, reversed without net harm. Whether a medicine identified as questionable is in fact reviewed, discussed and stopped depends on the behaviour of prescribers, the organisation of care and the participation of patients, and on each of these counts deprescribing is still done less often than the evidence would support.

The main barriers concern the organisation of care, professional responsibility and communication, not a lack of information. In a systematic review of forty studies across fourteen countries, Doherty et al. grouped them across cultural, organisational, interpersonal and individual levels [65]. At the cultural and organisational levels, they identified a clinical culture oriented towards diagnosing and prescribing rather than stopping, guidelines written around single diseases with little to say about multimorbidity, fragmented care in which prescribers in different settings neither communicate nor collaborate, and an absence of shared decision-support tools [65]. At the interpersonal and individual levels, they described limited consultation time, uncertainty among prescribers and patients about the consequences of stopping, and a professional reticence about altering a colleague’s prescription [65]. Some of these, including single-disease guidance, missing tools and the difficulty of seeing the whole list, are frictions that explicit criteria and decision support are designed to reduce. Others, such as professional etiquette and the working relationships between prescribers in different settings, will not yield to better software alone. These barriers do not fall evenly. The same review identified health inequalities in access to safe deprescribing for patients with sensory or cognitive impairment, for patients with mental illness and for patients from migrant communities, with older age combined with lower education a significant barrier in its own right [65].

Fragmentation is sharpest when a patient moves between settings. A systematic review of 24 randomised trials in 17,664 older adults found that interventions bridging the discharge transition, rather than acting at a single point, were the most likely to succeed. Pooled by component, self-management education or coaching (RR 0.81, 95% CI 0.74–0.89), telephone follow-up (0.84, 0.73–0.97) and medication reconciliation (0.88, 0.81–0.96) were each associated with fewer readmissions, whereas timely communication between sectors was not (0.90, 0.79–1.02) [66]. The authors are cautious about what this establishes, since variability between the trials was substantial, the components were coded by judgement from published descriptions, and no effect can be attributed to a single activity within a bundle. One trial of home-based medication review after discharge reported more readmissions in the intervention arm than under usual care [66]. One of the trials that did show benefit required an average of 114 min of pharmacist time per patient [66].

The same literature points to what helps: guidance on multimorbidity that says when and how to stop and not only when to start, interprofessional working with pharmacists, clear communication, and a patient-centred approach built on shared decision-making [65]. There is system-level evidence that these measures affect prevalence, not only individual regimens. Among the SHARE countries, the minority that reduced polypharmacy between waves did so against a background of measures of this kind, with physician training, electronic decision support and pharmacist-led interventions in France the clearest example (Figure 1) [11]. How much of that difference the measures explain is not established. The source describes the association without testing it, and a competing explanation runs through the same interval, since the two waves straddle the COVID-19 pandemic, during which access to care deteriorated for older adults and unmet need may have aggravated the conditions that drive prescribing [11]. Where prevalence rose, the same authors point to longer survival and population growth, and to the absence of national medication-review programmes in most of Europe [11]. They also caution that survey participants tend to be healthier and better motivated than the populations they represent [11]. No published analysis attributes a national change to a particular policy, and none examines whether pharmacogenomic or otherwise individualised prescribing has yet affected prevalence at this scale. That such measures coincided with falling national prevalence, while most countries rose, shows that detection from routine data becomes useful only when paired with a delivery mechanism.

Patient engagement remains the most underused of these mechanisms. Older adults are, on the whole, more willing to stop medicines than is often assumed. In a meta-analysis of twenty-nine studies and 11,049 participants, 87.6% (95% CI 83.3–91.4) said they would be willing to have a medication deprescribed if their doctor suggested it [64]. Willingness among caregivers was lower and far less certain (74.8%, 95% CI 49.8–93.8) [64]. The same authors caution that stated willingness predicts actual deprescribing poorly, so it has to be elicited in the individual encounter rather than assumed [64]. In the EMPOWER trial, a mailed, plain-language brochure setting out the harms of benzodiazepines and a tapering schedule led 27% of long-term users to discontinue at six months, against 5% receiving usual care, largely by prompting 62% of recipients to raise the subject with their physician or pharmacist [56]. Its success came from activating patients directly, so that the change began with them. Table 3 pairs each of these barriers with what the evidence supports doing about it and with what that measure does not resolve.

The pattern across barriers, facilitators and patient attitudes is consistent. The final decision, and the patient’s agreement to it, still require individual assessment that no screen can provide. The computational identification of candidates narrows down a population to those worth reviewing. Engaging the patient then helps ensure that the review ends in a decision the patient will make.

## 10. Conclusions

The evidence does not identify the number of medicines as the principal cause of the harm attributed to polypharmacy. Associations with mortality, hospitalisation and falls attenuate once comorbidity and high-risk drugs are accounted for, and deprescribing trials have lowered medication counts without reliably improving these outcomes. The clinically meaningful target is the appropriateness of the regimen and the burden it imposes, for which the drug count is a rough and partly confounded proxy.

Pharmacological burden can be derived from routinely collected data, and indices such as the Drug Burden Index apply directly to dispensing records. The explicit criteria apply to the same records wherever the clinical detail they call for is also held, so potentially inappropriate prescribing can be counted across populations. The decision to act is not computable. Whether a flagged medicine should be stopped for a particular patient, and whether that patient agrees, depends on clinical judgement, and this is where automated tools have performed the worst. Computation can identify the regimens that warrant a closer look, and clinicians and patients make the decision.

This is a narrative review. Its sources were selected purposively and do not represent an exhaustive appraisal of the field, and the criteria and burden measures on which it draws, though well established, remain imperfect proxies for the clinical judgement they are meant to support.

For practice, this implies structured, patient-centred medication review prioritised by pharmacological burden and appropriateness, with patients encouraged to raise the question themselves. Future trials should test whether triage by computable burden improves patient outcomes, measure frailty at baseline rather than age alone, and report the withdrawal schedule used. None of this touches the organisational barriers that will decide whether better detection reaches the patients who need it.

## Figures and Tables

**Table 1:** Instruments for assessing prescribing appropriateness in older adults.

Instrument (Version, Year)	Developer/Country	Method	Structure & Size	Targets	Application	Validation/Implementation	Key Limitation
AGS Beers Criteria (2023) [44]	American Geriatrics Society, New York, NY, USA	Explicit; expert panel + evidence review	5 categories (avoid; drug–disease/syndrome; use with caution; drug–drug interactions; renal dose-adjustment) + strong-anticholinergic list	PIMs, drug–disease, DDIs, renal	Reference avoidance list applied to a medication list	Most-cited PIM tool; basis of countless prevalence studies	US-centric formulary; some agents not marketed elsewhere
STOPP/START v3 (2023) [45]	O’Mahony et al., Europe (Delphi panel)	Explicit; Delphi consensus	190 criteria (133 STOPP + 57 START); organised by physiological system; +two-thirds vs. v2	PIMs and prescribing omissions	Rule list for medication review	Trialled in OPERAM and SENATOR	Modest yield without pharmacist input
EURO-FORTA v2 (2023) [46]	Pazan, Wehling et al.; 7 European countries	Explicit; Delphi + clinical validation	267 entries across 27 indications; graded A–D	Beneficial and inappropriate drugs	Positive/negative labelling per indication	Multi-country consensus validation	Indication-based; needs diagnosis context
PRISCUS 2.0 (2023) [47]	Mann et al., Germany	Explicit; update of the 2010 list	187 substances; per-drug alternatives and precautions	PIMs (national formulary)	National avoidance list	≈one-quarter of older Germans exposed to ≥1	Country-specific; limited transferability
STOPPFrail v2 (2021) [48]	Curtin, Gallagher, O’Mahony; Ireland	Explicit; Delphi consensus	25 criteria	PIMs in advanced frailty/limited life expectancy	Deprescribing checklist + shared decision-making	Validated near end of life	Narrow scope by design
Medication Appropriateness Index (MAI) (1992) [42,43]	Hanlon et al., USA (Duke)	Implicit; judgement-based instrument	10 questions per drug (indication, effectiveness, dose, correct and practical directions, drug–drug and drug–disease interactions, duplication, duration, cost); each rated A/B/C → weighted summated score 0–18	Overall appropriateness, incl. effectiveness, duration and cost	Rater scores each drug → index	Long-standing implicit standard; good to excellent reliability; used as an RCT outcome	Time-consuming; rater-dependent
Drug Burden Index (DBI) (2007) [17]	Hilmer et al., Australia/USA	Quantitative burden index; dose–response formula, not a judgement	Continuous score = cumulative anticholinergic + sedative load, dose-weighted	Functional drug burden (not a list)	Computed from dispensing data	Health ABC: higher DBI ↔ worse physical and cognitive function	Covers only two pharmacological actions

PIM = potentially inappropriate medication; DDI = drug–drug interaction. The instruments fall into three groups: explicit criteria (international, national and frailty-specific), implicit judgement-based instruments, and quantitative burden indices. The first two assess prescribing against a standard, whereas the third measures exposure and makes no judgement of appropriateness. Pooled prevalence of PIM use in older adults: 36.7% (94 studies, ~371 million people, 17 countries).

**Table 2:** A staged framework for medication review at population scale. Each stage lists the data it requires, who would carry it out, what it produces and what it cannot do.

Stage	Data Required	Carried out by	What It Produces	What It Cannot Do
1. Population screen	Dispensing register alone	Automated; no clinical contact	Drug Burden Index, sequences suggesting a prescribing cascade, and criteria that rest on the medication list alone	Does not distinguish appropriate from inappropriate use. Only 55% of flagged cascade cases were genuine on record review [29], and more than half of software-generated recommendations were judged clinically unimportant [45]
2. Prioritisation	Burden score, number of criteria flagged, age	Automated	A ranked list of patients for review	Does not predict who will benefit. No model exceeded an area under the curve of about 0.62 [61]
3. Clinical review	Diagnoses, laboratory results, treatment indication, prognosis	Pharmacist and physician together	A patient-specific assessment of each flagged medicine	Does not scale. The OPERAM intervention required roughly 97 min per patient [54]
4. Decision and patient agreement	The patient’s care goals and preferences	Clinician with the patient	An agreed change, implemented one drug at a time with monitoring [18]	Cannot be read from routine data. Stated willingness to deprescribe predicts actual deprescribing poorly [64]
5. Follow-up	Subsequent dispensing plus clinical assessment	The treating physician	Detection of withdrawal effects and of substitution by another hazardous drug	Cannot be omitted. Withdrawal carries a measurable physiological cost [57], and benzodiazepines were sometimes replaced by equally hazardous sedatives [56]

Potentially inappropriate prescribing is increasingly detectable in routinely held data, but establishing whether a flagged prescription is in fact inappropriate for the patient, and acting on it, still demands clinical judgement and time. A screen that triages a population by computable burden, and reserves scarce expert review for those it identifies, is where these constraints lead.

**Table 3:** Barriers to deprescribing and the measures the evidence supports against each. Each row pairs a barrier with what the reviewed evidence supports doing about it and with what that measure does not resolve.

Barrier	Level	What the Evidence Supports Doing	What It Does Not Resolve
Clinical culture oriented towards diagnosing and prescribing rather than stopping	Organisation	Guidance that treats stopping as part of prescribing, saying when and how to withdraw a drug and not only when to start it [65], applied through an explicit protocol: ascertain every drug and its indication, gauge the risk of drug-induced harm, weigh each drug’s benefit against its burden, prioritise, and withdraw one drug at a time with monitoring [18]	Does not create the clinician and pharmacist time a full review takes [54]
Guidelines written around single diseases, with little on multimorbidity	Organisation	Criteria developed for older adults with multimorbidity rather than for one disease, covering undertreatment as well as overtreatment [45], graded by indication [46], or narrowed to advanced frailty and limited life expectancy [48]	No list is definitive, and the realistic standard is regional validity rather than universality [44,47]
Fragmented care, with no clinician owning the whole medication list	Organisation	Medication reconciliation, associated with fewer readmissions (0.88, 95% CI 0.81–0.96), in a meta-analysis stratified by intervention component. Interventions that bridged the transition for up to 90 days were the most likely to succeed [66]	No effect can be attributed to a single component of a bundle, and one trial of home-based review after discharge reported more readmissions than usual care [66]
Absence of a shared decision-support tool	Organisation	Explicit criteria coded against linked administrative data, so that the whole list is visible without a clinical encounter [59], with a computable burden measure used to rank patients for review [17]	Decision support delivered without individual tailoring showed no effect on mortality [53], and prediction from routinely held data alone remains weak [61]
Limited consultation time	Organisation and prescriber	Interprofessional working, with a pharmacist carrying the structured part of the review and the physician the decision [65], and triage so that scarce review time reaches the patients a computable screen identifies (Table 2)	Does not remove the constraint. Pharmacists have more time for review but less knowledge of the individual patient, and need access to the practice record, which is not routine [65]. One effective transitional-care trial required an average of 114 min of pharmacist time per patient [66]
Uncertainty about the consequences of stopping	Prescriber and patient	Withdrawal one drug at a time with monitoring [18], and follow-up designed to detect withdrawal effects and substitution by another hazardous drug [56,57]. Pooled evidence found adverse drug-withdrawal events not significantly increased [53]. Prescribers reported that guidance on the risks and benefits of stopping, and discussion of difficult cases with experienced colleagues, improved their confidence in acting [65]	That estimate is imprecise and drawn from three studies, and the withdrawal schedule was not described in 23 of 37 randomised trials [53]. Withdrawing an antihypertensive carried a measurable physiological cost [57]. The uncertainty is not only clinical, since prescribers also reported fear of legal repercussions and of appearing to disengage from the patient [65]
Reticence about altering a colleague’s prescription	Prescriber	Continuity of care not only between clinician and patient but between the general practitioner, the pharmacist and the specialists involved, together with discussion of difficult cases with experienced colleagues, which prescribers reported as improving their confidence in acting [65]	Relational rather than informational. Timely communication between sectors on its own was not associated with fewer readmissions (0.90, 95% CI 0.79–1.02) [66]
Assumed patient reluctance	Patient	Ask rather than assume, since prescribers did not always seek their patients’ views [65] and 87.6% (95% CI 83.3–91.4) said they would accept deprescribing if their doctor suggested it [64]. Written information addressed to the patient can open the conversation, and a mailed brochure prompted 62% of recipients to raise long-term benzodiazepine use with their physician or pharmacist [56]	Stated willingness predicts actual deprescribing poorly and has to be elicited in the individual encounter [64]
Caregiver reluctance	Caregiver	Elicit the caregiver’s view alongside the patient’s rather than inferring it, since willingness among caregivers was lower and far less certain (74.8%, 95% CI 49.8–93.8) [64]. Prescribers depend on caregivers for patients with cognitive impairment and report withdrawing a recommendation after a relative objects, so managing caregivers’ expectations and conveying the reasons for a change belong in the plan [65]	The estimate is imprecise. In the transitional-care trials, caregivers were involved mostly as a source of information rather than as a target of the intervention, and the reviewers call for work identifying where they could be engaged more widely [66]
No non-pharmacological alternative to offer in place of the drug	Organisation	Access to the alternatives that make stopping possible, among them talking therapies where a psychotropic is being withdrawn. Prescribers named their absence as a reason for continuing and their availability as a facilitator [65]	Provision lies outside the prescriber’s control and is itself unevenly distributed [65]

The measures in the third column are drawn from the studies discussed in Section 7 and Section 9 and are not new recommendations. Each is paired with the limitation the same evidence records.

## Data Availability

Data sharing is not applicable to this article as no new data were created or analysed in this study.

## Supplementary Materials

The following supporting information can be downloaded at: https://www.mdpi.com/article/10.3390/geriatrics11040100/s1, Table S1: country-level prevalence of polypharmacy in the SHARE survey for the eighteen countries and regions covered by both wave 6 and wave 9, with the change in percentage points for each [10,11]. File S1: completed SANRA form for this review [19].
