# Supplementary material for: Appropriateness over Numbers: Pharmacological Burden and Scalable Medication Review in Older Adults
Source: Geriatrics (Basel). 2026 Aug 10;11(4):100. doi: 10.3390/geriatrics11040100 (PMC13512173; doi:10.3390/geriatrics11040100)
Supplement: Supplementary file 1 [file geriatrics-11-00100-s001.zip › geriatrics-4447276-supplementary.pdf]

Supplementary Materials

Appropriateness Over Numbers: Pharmacological Burden and Scalable Medication Review in Older Adults

Michał Pstrągowski, Dawid Hachlica, Łukasz Izbicki — Manuscript ID geriatrics-4447276

File S1. Completed SANRA form

The Scale for the Assessment of Narrative Review Articles (SANRA) comprises six items, each scored from 0 to 2. The review was scored by the authors against the revised manuscript. Page and line numbers refer to the revised version.

Item	Score	Justification	Location
1. Justification of the article's importance for the readership	2	Section 1 establishes that polypharmacy is common and rising, gives prevalence across eighteen European countries and two national time series, and states why a count-based framing is inadequate for clinical practice.	p. 2–5, ll. 34–125
2. Statement of concrete aims or formulation of questions	2	Section 1 states two linked claims explicitly and says what the review will ask on that basis. The aims paragraph is preceded by definitions of the three concepts the claims use.	p. 5, ll. 126–149
3. Description of the literature search	2	Section 2 names the databases, the search period of January to June 2026, the hand-searching of reference lists, the iterative handling of search terms, the eligibility criteria and the rule used to prioritise sources. This item scored 1 before revision, when search dates were not given.	p. 6–7, ll. 156–184
4. Referencing	2	Every empirical claim carries a citation to a primary source. The reference list holds 66 entries, of which the majority were published from 2020 onward. Statements drawn from instruments and criteria cite the source publications rather than secondary reviews.	p. 29–34
5. Scientific reasoning	2	Evidence against each of the review's two claims is reported alongside evidence for them, including cohorts in which the association with the drug count survived adjustment, and trials in which deprescribing produced no benefit or measurable harm. Where the authors of a pooled analysis reported heterogeneity or the certainty of their findings, these are given with the estimate.	p. 7, ll. 178–184; passim
6. Appropriate presentation of data	2	Effect estimates are given with confidence intervals throughout. Figure 1 presents prevalence from a single standardised source with its provenance stated in the caption and country-level values supplied as Table S1. Tables 1 and 2 pair each instrument and each stage with its documented limitation.	p. 3, ll. 75–89; Tables 1–3
**Total**	**12**	Maximum attainable score is 12.	

Reference for the instrument: Baethge, C.; Goldbeck-Wood, S.; Mertens, S. SANRA — a scale for the quality assessment of narrative review articles. Research Integrity and Peer Review 2019, 4, 5.

Table S1. Prevalence of polypharmacy by country, SHARE waves 6 and 9

Prevalence of the concurrent use of five or more medicines among adults aged 65 or older, for the eighteen countries and regions covered by both waves, as published in the source cited as reference [11] in the manuscript, where the values are standardised for age and sex to the 2013 European standard population. Countries are ordered by the change between waves. The final row gives the unweighted mean across these eighteen countries, which is the average line plotted in Figure 1. No values were read from published figures and no reanalysis was performed other than this average.

**Country or region**	**Wave 6 (2015), %**	**Wave 9 (2021–22), %**	**Change, percentage points**
Poland	33.8	51.8	+18.0
Portugal	36.9	51.3	+14.4
Israel	37.5	51.5	+14.0
Denmark	32.7	39.6	+6.9
Sweden	31.0	37.8	+6.8
Czechia	39.9	46.6	+6.7
Croatia	27.3	32.7	+5.4
Estonia	28.4	33.5	+5.1
Belgium	34.0	38.2	+4.2
Spain	31.6	33.8	+2.2
Germany	30.3	32.1	+1.8
Italy	32.9	32.7	−0.2
Austria	31.5	31.2	−0.3
Switzerland	26.3	26.0	−0.3
Luxembourg	29.5	29.2	−0.3
France	31.0	28.7	−2.3
Slovenia	28.1	25.0	−3.1
Greece	29.3	25.9	−3.4
**Unweighted mean, 18 countries**	**31.8**	**36.0**	**+4.2**

Eleven of the eighteen countries and regions rose between the two waves and seven fell.
